# Supplementary material for: Thalamocortical dynamics underlying spontaneous transitions in beta power in Parkinsonism
Source: Neuroimage. 2019 Jun;193:103–14. doi: 10.1016/j.neuroimage.2019.03.009 (PMC6503152; doi:10.1016/j.neuroimage.2019.03.009)
Supplement: Supplementary file 1 [file mmc1.docx]

**Supplementary Materials**

**Title:** **Thalamocortical dynamics underlying spontaneous transitions in beta power in Parkinsonism**

**Authors:** Carolina Reis^1,2^, Andrew Sharott^1^, Peter J. Magill^1,5^, Bernadette van Wijk^3,4^, Thomas Parr^3^, Peter Zeidman^3^, Karl Friston^3^, Hayriye Cagnan ^1,2^

1. Medical Research Council Brain Network Dynamics Unit, University of Oxford, Oxford, UK; 2. Nuffield Department of Clinical Neurosciences, John Radcliffe Hospital, University of Oxford, Oxford, UK; 3. Wellcome Centre for Human Neuroimaging, University College London, UK; 4. Integrative Model-based Cognitive Neuroscience Research Unit, University of Amsterdam, the Netherland; 5. Oxford Parkinson’s Disease Centre, University of Oxford, Oxford, UK.

**Corresponding author at:** MRC Brain Network Dynamics Unit at the University of Oxford, UK

E-mail address: hayriye.cagnan@ndcn.ox.ac.uk (Hayriye Cagnan)

Throughout this study, we were interested in synaptic modulation in the TC loop during beta enhancement in spectral data from 6-OHDA-lesioned rats. This was motivated by recent PD literature defining beta oscillations as intermittent events where power waxes and wanes within a time window of a second. To study the dynamic properties of this parkinsonian biomarker, many of the studies in the field focus on a binary characterization of the beta signal in the PD clinical context while using the envelope of the beta filtered signal and an arbitrary threshold of its mean to divide beta into physiological epochs and hypothetically pathological epochs (Feingold et al., 2015; Sherman et al., 2016; Tinkhauser et al., 2017; Little et al., 2013). Some studies have gone further and suggest that the duration of such high beta events is positively correlated with PD motor impairments.

In CSD-DCM, conditions are effectively spectral densities derived from segments of the data one is interested in studying and as such three considerations must be taken into account: 1) the oscillatory activity undergoing a PSD analysis must have a duration of at least two cycles of its frequency (approximately more than 130 msec for beta frequencies); 2) conditions must consistently show different power levels across subjects and 3) PSD analysis does not account for the time evolution of beta power (does not distinguish segments with short vs. long bursts).

Extracting 500 msec segments (>130msec) with extremely high/low sustained levels of beta power (area below the envelope) allowed us to obtain non-overlapping conditions across subjects with a satisfactory frequency resolution and different functional features: low beta hypothetically accounting for a physiological coupling state and high beta possibly accounting for a state with a higher probability of pathological coupling state.

Ad hoc beta burst analysis within conditions

Although we were unable to model beta bursts using CSD-DCM, we analysed the burst density in both low and high beta conditions a posteriori. Making use of the 75% percentile of the mean envelope as a threshold, the mean number of bursts in the LB condition (across trials and subjects) was 0.9 ± 0.3 and those bursts had the mean duration of 195 ± 106 msec. The mean number of bursts in the HB condition was 3.9 ± 0.7 and had the mean duration of 772 ± 188 msec. As expected, not only HB conditions show a higher density of beta bursts than LB conditions (approximately 4:1 in our data) but also its bursts show a longer duration. In both conditions, some trials showed incomplete bursts that were still included in the computation of average number of bursts and averaged duration of bursts per condition.

Fig.S.1. – Beta conditions and beta bursts. Example of Beta bursts analysis in the Low and High beta conditions. In this example we can observe how low beta (in blue) and high beta (in red) have different levels of power and present different number of beta bursts. LB with no beta burst and HB with 3 beta bursts.

Fig.S.2. – FFX-BMC analysis of the 144 models. The bar chart shows normalised F values for each model. From the 144 models (9 architectures x 16 modulatory configurations), model number 134 (orange) was the one with the higher log-evidence value (3.6649x10^4^) showing a difference of approximately 6 from model 133 (beige) whose log-evidence was the second highest (3.6643x10^4^). Second most plausible model being the model with changes in effective connectivity among the following subpopulations: superficial and deep pyramidal cells reciprocally, relay and reticular cells reciprocally, relay cells to deep pyramidal cells and inhibitory interneurons and deep (Illustrated in Fig.3 - Factor 1: Architecture, number 9; Factor 2: Modulatory configuration, number 5). Note that the above models are identical in their synaptic network except for the intracortical modulatory connections – while the winning model allows cortical modulatory dynamics between superficial and middle pyramidal populations, the alternative model shows intracortical connectivity changes between superficial and deep pyramidal cells. Nonetheless, both models highlight a change in glutamatergic inputs to the superficial pyramidal cells via intrinsic (and fast) interactions as a contributing factor for beta power enhancement.

From the averaged posterior estimations on parameters derived from PEB (Fig.6), we computed the net activity of each subpopulation during both LB and HB conditions. The same procedure has been applied to the remaining models and can be found in the Supplementary section in (Fig.S.4). This analysis was performed by weighting the combined coupling strength from different efferent subpopulations to an afferent subpopulation; i.e., by using the fixed parameter values of intrinsic and extrinsic connections and the posterior estimations of A/G parameters in the baseline condition and A/G plus B parameters in the HB condition. As observed in the bar plot below (Fig.S.3), the net-activity of our neural mass model presents similar results in both conditions: SP, DP, II and REL receiving a stronger excitatory input than inhibitory (net-excited) and MP and RET receiving a stronger inhibitory input than excitatory (net-inhibited). It is worth noting that to induce a transient beta increase all subpopulations increased their baseline level of activation, i.e., subpopulations that were net-excited in the low condition received a stronger excitatory drive in the high condition and subpopulations that were net-inhibited in the low condition received further inhibitory inputs during the high condition. This effect is most predominantly observed in SP, followed by MP and DP.

Figure.S.3 – Net-activity state of subpopulations. Overall activation state of neural mass model subpopulations in the different conditions. Bar plot of net-activity of subpopulations in the LB condition (blue) and high beta condition (red). Of interest is the comparison of net-activation levels of each subpopulation in the different conditions and consequently noting that SP is the subpopulation that shows a higher alteration in net-activity (followed by MP and DP).

Fig.S.4. – Net-activity state of subpopulations at low and high beta. Comparing the net-activation pattern across the winning generative model and the average of all models (n=144), we can observe a similar directionality of effects across populations except for the middle pyramidal subpopulation. Also, the mechanism of the created state space (independently of the modulatory pathways) to achieve the spectral transition in our data is consistent between the two plots - an intensification of the baseline level of activation.


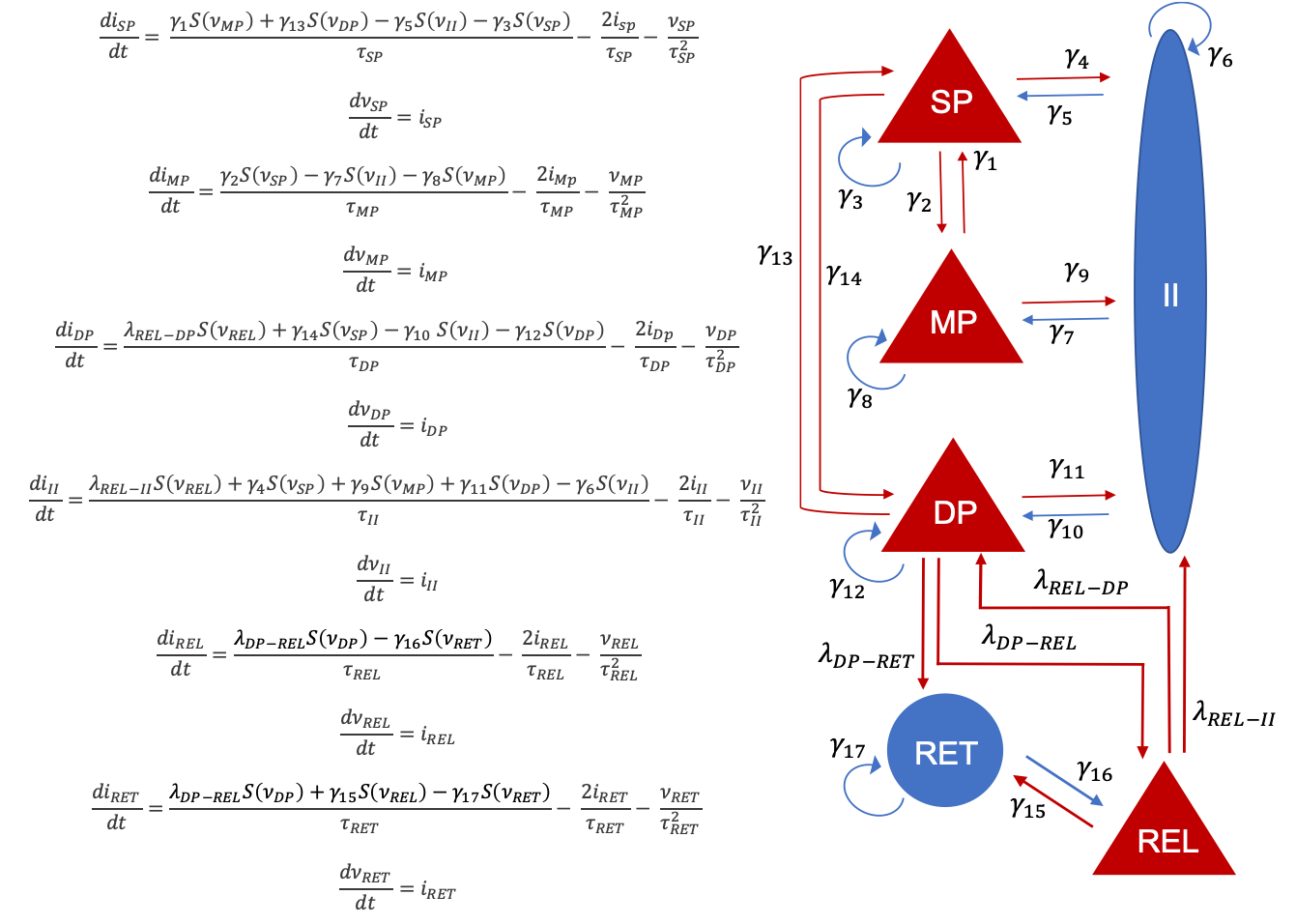


Fig.S.5 – Differential equations of the winning model. i -currents; $\boldsymbol{v}$- voltages; SP/MP/DP- middle/superficial/deep pyramidal cells; II -inhibitory interneurons; REL – thalamic relay cells; RET – thalamic reticular cells; $\boldsymbol{\gamma}$ – synaptic coupling strength for intrinsic connections; 𝝀 - synaptic coupling strength for extrinsic connections; $\boldsymbol{S}$– sigmoid function; $\boldsymbol{\tau}$ – time constant. Blue coloured arrows denote GABAergic connections, while red arrows refer to Glutamatergic connections. Note that intralaminar coupling between pyramidal cells and local inhibitory interneurons ($\boldsymbol{\gamma}$3, $\boldsymbol{\gamma}$8, $\boldsymbol{\gamma}$12) is parameterised with a single effective connectivity and should be contrasted with the interlaminar coupling with the common pool of inhibitory interneurons ($\boldsymbol{\gamma}$5, $\boldsymbol{\gamma}$7, $\boldsymbol{\gamma}$10) (see Auksztulewicz and Friston, 2015).


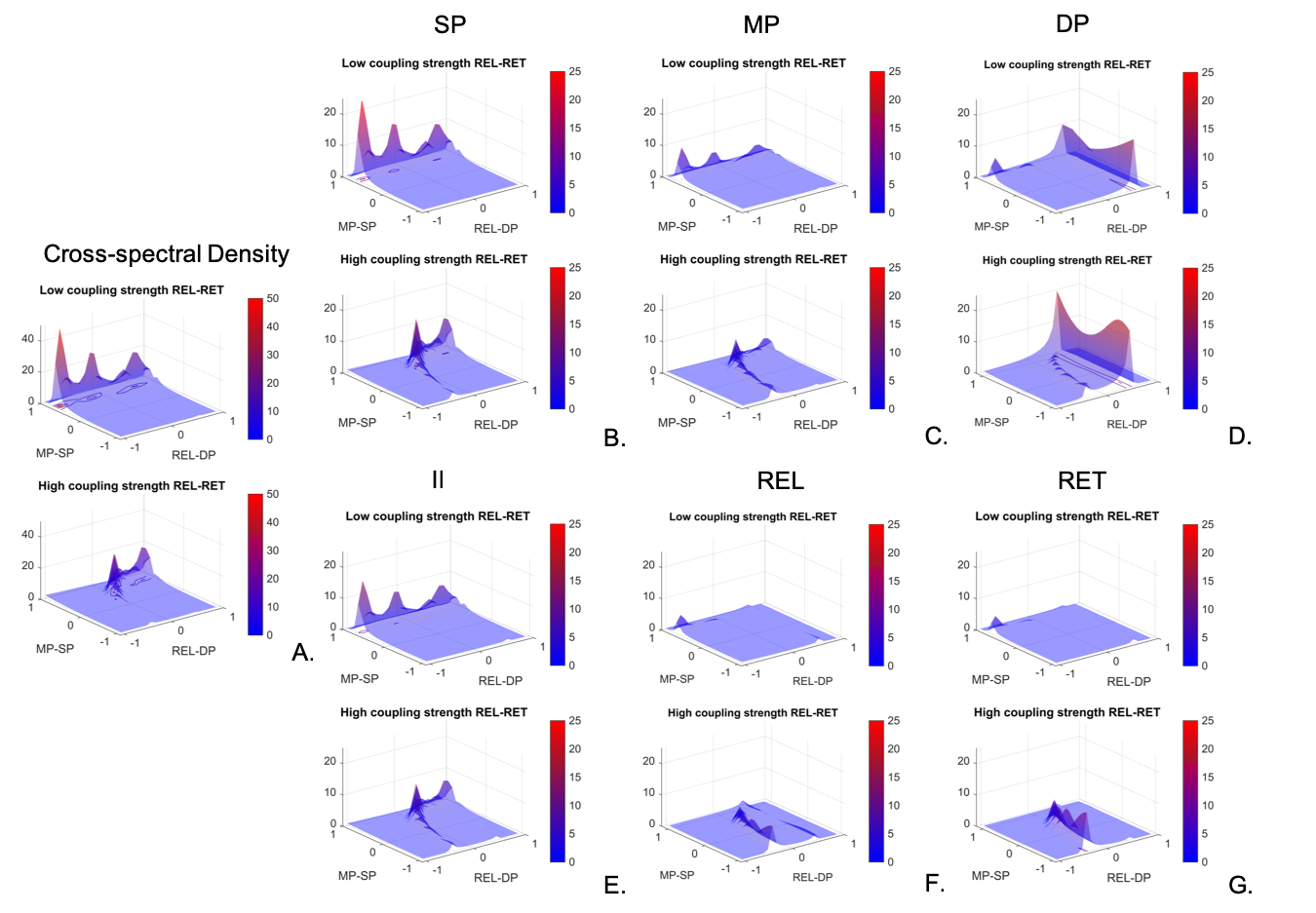
Fig.S.6- Exploration of parameters space of connections with the greatest effect on beta enhancement at the subpopulation level.

To explore the possibility that simplified models – with changes in inter-laminar excitatory-inhibitory coupling – could explain an increase in cortical beta activity, the following Bayesian model comparison was performed:


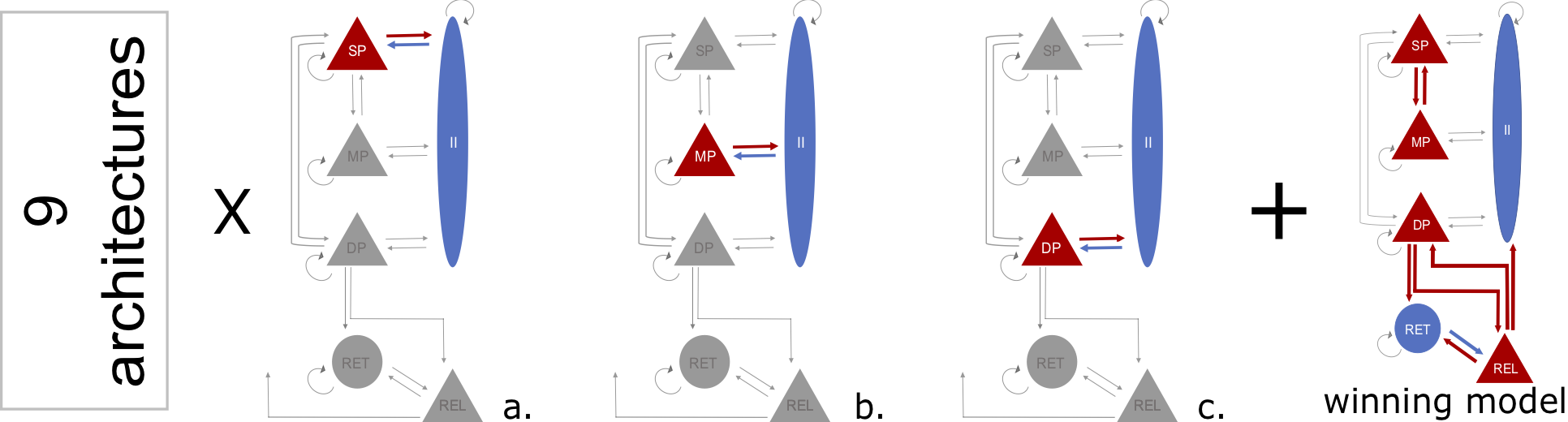


Fig.S.7 – Model space for a supplementary BMC, comparing models with changes in excitatory-inhibitory coupling (between pyramidal cells and the common inhibitory population) with the winning model. Here, 28 models are considered, the winning model plus 27 additional models. For each architecture shown in the top panel of Fig.3, condition specific changes in the following connections were considered: a) reciprocal connections between superficial pyramidal cells and the common inhibitory subpopulation; b) reciprocal connections between middle pyramidal cells and the common inhibitory subpopulation and c) reciprocal connection between middle pyramidal cells and the common inhibitory subpopulation.

The winning model remained the model identified in the main text (shown here as model number 28), with a free-energy difference of 1190 from the model with the second highest model evidence (model 6 – architecture 2 and modulatory configuration c). This result suggests that condition-specific effects in the winning model are the best explanation for the increase in cortical beta activity, in relation to changes in excitatory/inhibitory coupling between pyramidal cells and a common inhibitory population.


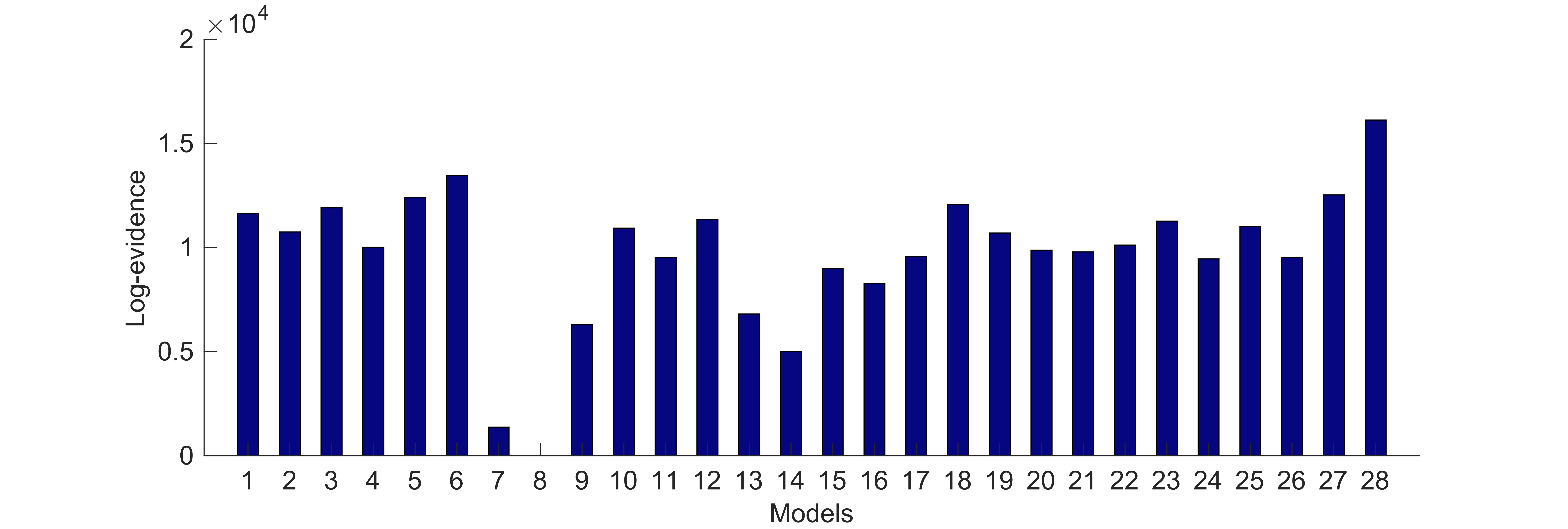
Fig.S.8 – FFX-BMC analysis of 27 additional models (plus the winning model). The bar chart shows normalised F values (i.e., log evidence) for each model.
